# Biopharma innovation trends during COVID-19 and beyond: an evidence from global partnerships and fundraising activities, 2011-2022

**DOI:** 10.1186/s12992-023-00953-6

**Published:** 2023-08-14

**Authors:** Tzu-Hui Yu, Yung-Yu Mei, Yufeng Jane Tseng

**Affiliations:** 1 Department of Economics, National Taiwan University, No. 1 Sec. 4, Roosevelt Road, Taipei, Taiwan; 2 Science & Technology Policy Research and Information Center, National Applied Research Laboratories, No. 106, Heping East Road, Taipei, Taiwan; 3 Department of Computer Science and Information Engineering, National Taiwan University, No. 1 Sec. 4, Roosevelt Road, Taipei, Taiwan; 4 Graduate Institute of Biomedical Electronics and Bioinformatics, National Taiwan University, No. 1 Sec. 4, Roosevelt Road, Taipei, Taiwan

**Keywords:** COVID-19, Biopharmaceutical industry, Fundraising, Partnership, Innovation

## Abstract

**Background:**

Co-development alliances and capital-raising activities are essential supports for biopharmaceutical innovation. During the initial outbreak of the COVID-19, the level of these business activities has increased greatly. Yet the magnitude, direction, and duration of the trend remain ambiguous. Real-time real-world data are needed to inform strategic redirections and industrial policies.

**Methods:**

This observational study aims to characterize trends in global biopharma innovation activities throughout the global pandemic outbreak. Our extensive deal dataset is retrieved from the commercial database GlobalData (12,866 partnership deals and 32,250 fundraising deals announced between 2011 and 2022). We perform Chi-squared tests to examine the changes in qualitative deal attributes during and beyond the outbreak. Our deal-level sample is further aggregated into category-level panel data according to deal characteristics such as therapy area, molecule type, and development phase. We run a series of regressions to examine how the monthly investment amount raised in each category changed with the onset of the pandemic, controlling for the US Federal funds rate.

**Results:**

The temporary surge of partnership and capital-raising activities was associated with the increase in infectious disease-related deals. Academic and government institutions played an increased role in supporting COVID-related co-development partnerships in 2020, and biopharma ventures had been securing more investments in the capital market throughout 2020 and 2021. The partnership and investment boom did not last till the later pandemic in 2022. The most significant and enduring trend was the shifting focus toward discovery-phase investments. Our regression model reveals that the discovery-phase fundraising deals did not suffer from a bounce back in the late pandemic, consistent with a persistent focus on early innovation.

**Conclusions:**

Despite the reduced level of partnership and fundraising activities during 2022, we observe a lasting change in focus toward biopharmaceutical innovation after the pandemic outbreak. Our evidence suggests how entrepreneurs and investors should allocate resources in response to the post-pandemic tight monetary environment. We also suggest the need for policy interventions in financing private/public co-development partnerships and non-COVID-related technologies, to maintain their research capacity and generate breakthroughs when faced with unforeseen diseases.

**Supplementary Information:**

The online version contains supplementary material available at 10.1186/s12992-023-00953-6.

## Background

Collaboration and capital investment are crucial for biopharmaceutical drug development, as early innovation is often driven by academic institutions or small biotech firms [1, 2]. Lacking resources, these relatively smaller entities seek partnerships with large corporations to increase capacity, marketing, and product improvement [2–4]. In addition, small biotech firms depend on the free flow of capital to support their ideas and generate clinical benefits [1].

The emergence of the COVID-19 pandemic signaled a scientific paradigm shift toward accelerated innovation [5]. Many technological advancements for COVID-19 vaccines were invented by academic labs or small biotech companies through co-development programs and licensing agreements with larger companies. These collaborations expedite the progress of creating next-generation vaccines, utilizing genomic vaccine and gene editing technologies. The innovative techniques hold the potential for combatting other diseases. However, the development and commercialization of these technologies are contingent on continued industry-academia collaboration. It remains unclear whether the pandemic brings expanded R & D innovation ecosystems and spillover effects to co-development activities in other therapeutic areas [6–8].

On the other hand, the growing attention to the biopharma industry during the pandemic may have played a role in providing capital for innovation [9–12].Some researchers and investors hold the belief that the pandemic has influenced long-term value-based investors to consider investing in the life sciences sector post-pandemic, with the expectation of enduring growth and prosperity [11, 13, 14] . However, other studies suggest the biopharma capital market would not continue to see such encouraging growth [15, 16].

In addition to the pandemic’s fundamental impact on biopharma fundraising activities, the fluctuation in deal amounts can also be attributed to the global monetary policies implemented during this period. Governments responded to the initial outbreak by swiftly adopting monetary easing policies to mitigate potential economic downturns. These policies involved measures such as expanding the money supply and reducing interest rates, which are conventional tools used to stimulate investments and support economies.

For instance, the US Federal Reserve cut the Federal funds rate from 1.58% to 0.65% in March 2020, marking the largest decrease since the 2008 financial crisis. The Federal funds rate serves as a target interest rate that influences market interest rates, exerting a substantial influence on global financial policies and economic performance. While this dramatic rate cut resulted in increased business investments on a global scale, it also gave rise to significant inflationary pressures, particularly within the domestic economy. In 2022, the US Federal Reserve dramatically hiked the Fed rates to lower market interest rates and fight inflation, resulting in reduced business spending and a more cautious approach from global investors.

Biopharmaceutical companies, in particular, are highly susceptible to rising interest rates, as they require continuous capital injections to support the lengthy drug development process [17, 18]. During the later stages of the pandemic, as public attention towards the biopharma sector waned, the global monetary tightening environment introduced additional uncertainties regarding future trends in biopharma innovation.

This study represents the first empirical analysis that examines the lasting transformations in biopharma innovation endeavors in the post-COVID-19 era. By employing an extensive dataset, we aim to address the following research questions: (i) How did the number of biopharma partnerships and the volume of fundraising deals evolve during and after the initial pandemic outbreak? (ii) Throughout the pandemic, did the industry experience a shift in its focus on specific therapy areas and molecule types? (iii) What are other underlying shifts in biopharma fundraising activities, while accounting for the changes in financial policies implemented in response to the pandemic? Given the tightened financial conditions experienced in 2022, what can we anticipate regarding the long-term trends in the fundraising activities of the biopharma industry?

## Methods

### Data sources

Our primary dataset consists of 12,866 partnership deals and 32,250 fundraising deals (including venture capital, private equity, and equity offering deals) announced between 2011 and 2022 in the global biopharmaceutical industry, collected from Pharma Intelligence Center of the commercial database GlobalData on March 30, 2023. GlobalData summarizes information on biopharma business deals–including fundraising and partnership deals–by capturing daily news updates through alerts from company websites, stock exchanges, trade, and external media sources. This Deals data set is further integrated with the GlobalData in-house Drugs, Companies, and Financials database. Our final dataset contains three categories of variables: deal attributes (e.g., announcement date, deal value, and deal type), drug attributes (e.g., therapy area and molecule type), and company attributes (e.g., issuing/acquiring company name and entity type). GlobalData covers all stages of deals across 24 therapy areas and 37 molecule types, indicating a diverse and heterogeneous sample. The database has been used in relevant studies that unravel the research and development dynamics in the pharma industry [19–21]. Detailed definitions and summaries of all variables are provided in Tables 1, 2, 3 and 4.^1^

**Table 1:** The composition of partnerships (infectious disease only)

		Average yearly count (%)^a^									
		2011-2017		2018-2019 (base group)		2020			2021-2022		
**Infectious Disease Partnership (N=3,001)**		**Avg yearly count: 227**		**Avg yearly count: 193**		**Avg yearly count: 518**			**Avg yearly count: 254**		
**Entity Type**^**b**^	The ownership of the target company										
Public	Involving only publicly owned companies	30	15%	48	25%	81	16%	*	56	22%	
Private	Involving only privately owned companies	35	18%	24	12%	81	16%	*	36	14%	
Public + Private	Involving both publicly and privately owned companies	59	30%	61	32%	159	31%	*	90	36%	
Missing	-	(30)		(4)		(3)			(0)		
**Region**	The geographical region where the deal took place										
Asia-Pacific	-	47	30%	51	32%	152	33%		78	36%	
Europe	-	42	26%	40	25%	91	20%		48	22%	
North America	-	54	34%	58	37%	175	39%		66	31%	
Others	-	17	11%	10	6%	36	8%		23	11%	
Missing	-	(67)		(34)		(64)			(39)		
**Molecule Type**^**c**^	With a drug/drug candidate of a specific molecule type										
Gene/Cell Therapy	-	9	5%	14	7%	27	5%		10	4%	
Protein	-	13	6%	11	6%	20	4%		16	6%	
Vaccine	-	43	22%	30	17%	170	34%	***	72	29%	**
Anti-Body	-	30	15%	48	26%	121	24%		56	22%	
Peptide	-	12	6%	10	5%	20	4%		6	2%	
Small Molecule	-	98	50%	88	48%	155	31%	***	102	41%	
Recombinant	-	16	8%	18	10%	48	10%		22	9%	
Biologic	-	3	2%	4	2%	9	2%		8	3%	
Oligonucleotide	-	8	4%	14	8%	33	7%		26	11%	

^a^ We use the pre-outbreak deals as the baseline group to perform chi-squared tests, examining whether the composition of partnership activities changed during or after the initial global outbreak. The significance of each chi-squared test is denoted by asterisks (*$P<0.05$, **$P<0.01$, ***$P < 0.001$).

^b^ This is a nominal variable, so the percentages of all subjects add up to 100%.

^c^ This is a set of binary variables that are not mutually exclusive and collectively exhaustive. Since companies have more than one drug in their portfolio, each company could be categorized into several therapy areas or molecule types. Since companies have more than one drug in their portfolio, each company could be categorized into several therapy areas or molecule types. To address the multiple comparisons issue, we adjust the significance levels per test through Holm’s step-down extension of the Sidak method, controlling the family-wise error rate at 0.05. The unadjusted significance levels are reported in eTable 4 in the online supplement

**Table 2:** The composition of partnerships (non-infectious disease)

		Average yearly count (%)^a^								
		2011-2017		2018-2019 (base group)		2020			2021-2022	
**Non-Infectious Disease Partnership (N=9,865)**		**Avg yearly count: 811**		**Avg yearly count: 790**		**Avg yearly count: 910**			**Avg yearly count: 849**	
**Entity Type** ^**b**^	The ownership of the target company									
Public	Involving only publicly owned companies	106	14%	138	18%	181	20%		158	19%
Private	Involving only privately owned companies	156	21%	130	17%	170	19%		169	20%
Public + Private	Involving both publicly and privately owned companies	261	36%	314	40%	349	39%		344	41%
Institution + Public/Private	Involving institutions and other companies	208	28%	200	26%	205	23%		176	21%
Missing	-	(80)		(8)		(5)			(2)	
**Region**	The geographical region where the deal took place									
Asia-Pacific	-	143	26%	198	33%	251	36%		245	36%
Europe	-	164	30%	147	25%	184	26%		158	23%
North America	-	184	34%	201	34%	205	29%		219	32%
Others	-	55	10%	49	8%	59	8%		58	8%
Missing	-	(265)		(195)		(211)			(169)	
**Therapy Area** ^**c**^										
Immunology	-	96	13%	92	12%	106	12%		90	11%
Oncology	-	326	45%	364	47%	465	51%		408	48%
Gastrointestinal	-	75	10%	90	11%	57	6%	***	76	9%
Musculoskeletal Disorders	-	53	7%	58	7%	49	5%		48	6%
Metabolic Disorders	-	95	13%	68	9%	63	7%		63	7%
Ophthalmology	-	54	7%	60	8%	57	6%		49	6%
Cardiovascular	-	58	8%	54	7%	54	6%		42	5%
Central Nervous System	-	177	24%	182	23%	160	18%	*	184	22%
Respiratory	-	50	7%	46	6%	51	6%		42	5%
Others	-	199	27%	216	28%	228	25%		233	28%
**Molecule Type** ^**c**^	With a drug/drug candidate of a specific molecule type									
Gene/Cell Therapy	-	93	13%	140	18%	170	20%		131	17%
Protein	-	51	7%	36	5%	41	5%		39	5%
Vaccine	-	23	3%	22	3%	18	2%		12	2%
Anti-Body	-	128	18%	146	19%	178	21%		175	23%
Peptide	-	38	5%	32	4%	36	4%		30	4%
Small Molecule	-	362	51%	355	47%	369	43%		350	45%
Recombinant	-	42	6%	27	4%	17	2%		19	2%
Biologic	-	13	2%	15	2%	17	2%		22	3%
Oligonucleotide	-	27	4%	28	4%	36	4%		29	4%

^a^ We use the pre-outbreak deals as the baseline group to perform chi-squared tests, examining whether the composition of partnership activities changed during or after the initial global outbreak. The significance of each chi-squared test is denoted by asterisks (*$P<0.05$, **$P<0.01$, ***$P < 0.001$).

^b^ This is a nominal variable, so the percentages of all subjects add up to 100%.

^c^ This is a set of binary variables that are not mutually exclusive and collectively exhaustive. Since companies have more than one drug in their portfolio, each company could be categorized into several therapy areas or molecule types. To address the multiple comparisons issue, we adjust the significance levels per test through Holm’s step-down extension of the Sidak method, controlling the family-wise error rate at 0.05. The unadjusted significance levels are reported in eTable 4 in the online supplement

**Table 3:** The composition of fundraising activities

		Average yearly amount - in US$ 100 million dollars (%)^a^									
		2011-2017		2018-2019 (base group)		2020-2021			2022		
**All Fundraising Deals (N=32,250)**		**Avg yearly amount: 767**		**Avg yearly amount: 1145**		**Avg yearly amount: 2615**			**Avg yearly amount: 1617**		
**Deal Subtype** ^**b**^	Stage of the financing cycle for the target company										
Early stage	Conceptualization stage with products not fully developed	42	5%	100	9%	181	7%	***	159	10%	***
Later stage	The product is fully developed, tested, and ready to be launched	73	10%	137	12%	335	13%	***	215	13%	***
Private equity	Acquired by a private equity firm	157	20%	183	16%	449	17%	***	186	11%	***
IPO	Initial public offering	119	15%	116	10%	426	16%	***	104	6%	***
Other equity offerings	Private investment in public equity or secondary offerings	376	49%	609	53%	1,224	47%	***	952	59%	***
**Region** ^**b**^	The geographical region where the deal took place										
Asia-Pacific	-	186	24%	210	18%	578	22%	***	289	18%	***
Europe	-	155	20%	214	19%	514	20%	***	165	10%	***
North America	-	402	52%	708	62%	1,489	57%	***	1,150	71%	***
Others	-	23	3%	14	1%	33	1%	***	11	1%	***
**All Fundraising Deals w/ Available Drug Attributes (N=17,044)**		**Avg yearly amount: 349**		**Avg yearly amount: 653**		**Avg yearly amount: 1466**			**Avg yearly amount: 673**		
**Development Phase** ^**b**^	The development stage of the least advanced drug/drug candidate in the portfolio of the target company										
Discovery	Identification and optimization of a substance for therapeutic use	72	21%	223	34%	684	47%	***	334	50%	***
Preclinical	Testing of a drug in nonhuman animals or in vitro studies for a candidate drug	113	32%	261	40%	439	30%	***	196	29%	***
Clinical	Testing of a drug in humans to determine health outcomes	99	28%	127	19%	271	19%	***	131	19%	***
FDA reviewed/Marketed	FDA-reviewed or marketed drugs	65	19%	42	6%	72	5%	***	12	2%	***
**Therapy Area** ^**c**^	With a drug/drug candidate in a specific disease area										
Metabolic Disorders	-	145	41%	230	35%	498	34%		212	29%	
Cardiovascular	-	116	33%	159	24%	378	26%		129	18%	
Immunology	-	133	37%	256	39%	631	43%		257	36%	
Oncology	-	202	57%	437	66%	1030	70%		437	61%	
Respiratory	-	111	31%	166	25%	428	29%		140	20%	
Musculoskeletal Disorders	-	103	29%	151	23%	381	26%		140	19%	
Infectious Disease	-	142	40%	250	38%	662	45%	*	284	39%	
Gastrointestinal	-	129	36%	182	28%	453	31%		182	25%	
Central Nervous System	-	172	49%	266	40%	633	43%		270	38%	
**Molecule Type** ^**c**^	With a drug/drug candidate of a specific molecule type										
Gene &Cell	-	45	13%	119	18%	357	25%	**	149	21%	
Protein	-	69	19%	97	15%	308	21%	**	111	16%	
Vaccine	-	31	9%	52	8%	169	12%		76	11%	
Anti-Body	-	69	19%	167	25%	506	35%	***	216	31%	
Peptide	-	50	14%	71	11%	190	13%		67	10%	
Small Molecule	-	241	68%	382	58%	839	58%		382	54%	
Recombinant	-	73	21%	95	14%	244	17%		93	13%	
Biologic	-	7	2%	17	3%	107	7%	***	41	6%	
Oligonucleotide	-	22	6%	47	7%	154	11%		45	6%	

^a^ We use the pre-outbreak deals as the baseline group to perform chi-squared tests, examining whether the composition of fundraising activities changed during or after the initial global outbreak. The significance of each chi-squared test is denoted by asterisks (*$P<0.05$, **$P<0.01$, ***$P < 0.001$). No missing values for all variables listed in the table.

^b^ This is a nominal variable, so the percentages of all subjects add up to 100%.

^c^ This is a set of binary variables that are not mutually exclusive and collectively exhaustive. Since companies have more than one drug in their portfolio, each company could be categorized into several therapy areas or molecule types. To address the multiple comparisons issue, we adjust the significance levels per test through Holm’s step-down extension of the Sidak method, controlling the family-wise error rate at 0.05. The unadjusted significance levels are reported in eTable 5 in the online supplement

**Table 4:** Summary statistics of quantitative variables in fundraising deals

	Category-level	Deal-level
	Monthly Amount in 100 Million USD	Avg yearly Value in Million USD
	coef/(std)	coef/(std)
Fed rate	-0.222**	-0.456*
	(0.08)	(0.22)
Early	0.927***	0.457
	(0.24)	(0.59)
Late	-1.412***	-1.964*
	(0.29)	(0.80)
Discovery	0.111	-0.341
	(0.17)	(0.35)
Early x Discovery	0.994***	2.428***
	(0.25)	(0.62)
Late x Discovery	1.206***	1.655
	(0.34)	(0.93)
Linear Time	0.002***	0.004***
	(0.00)	(0.00)
Season (Q2)	-0.020	0.186
	(0.13)	(0.35)
Season (Q3)	0.123	1.209***
	(0.13)	(0.36)
Season (Q4)	-0.023	0.346
	(0.13)	(0.35)
Constant	0.462	5.266***
	(0.28)	(0.59)
sigma_u		
Constant	1.350***	
	(0.18)	
sigma_e		
Constant	3.069***	
	(0.03)	
R Squared		0.01
N of cases	4608	15302

$$p<0.05$$; **$$p<0.01$$; ***$$p<0.001$$

Additionally, monthly data on the US Federal funds rate from 2011 to 2022 are retrieved from FRED, Federal Reserve Bank of St. Louis [22]. We use this rate as a proxy for the global monetary easing environment - a lowered Fed rate tends to lower interest rates in capital markets, indicating more rigorous monetary easing measures. Its use as a proxy is supported by relevant literature [23–25].

### Descriptive and inferential statistics

Our first set of analyses explores raw trends in the composition of biopharma partnership deals and fundraising deals.

In the partnership-activity analysis, deals are divided into different categories based on several characteristics, including *EntityType* (ownership of the companies involved in the partnership), *Region* (the geographical region where the deal took place), *TherapyArea* (a set of binary variables indicating whether the involved companies have a drug/drug candidate in a specific disease area), and *MoleculeType* (a set of binary variables indicating whether the involved company has a drug/drug candidate of a particular type of molecule). We report the annual deal counts of different categories in four periods: 2011-2017, 2018-2019 (baseline group), 2020, and 2021-2022. Pearson chi-squared tests are performed to examine whether the deal composition changed during the 2020 and 2021-2022 periods, compared to the baseline group^2^.

Note that *TherapyArea* and *MoleculeType* are sets of binary variables, instead of normal categorical variables such as *EntityType*, since companies have more than one drug in their portfolio, each company could be categorized into several therapy areas or molecule types. As there are many comparisons between the different categories in *TherapyArea* and *MoleculeType*, the multiple comparisons issue might arise − leading to potentially inflated p-values and undermining the robustness of our results. To address the issue, we adjust the significance levels per test through Holm’s step-down extension of the Sidak method^3^, controlling the family-wise error rate at 0.05 [26]. The unadjusted results are reported in the online supplement (eTables 4 and 5) for scrutiny.

As for the fundraising activities, we perform similar Pearson chi-squared tests to examine changes in categorical characteristics. Yet the analyses here use slightly different outcome measures and definitions of variables and periods.

First, to better reflect the shifts of investment focus, we present the percentages of the total annual amounts^4^ raised in each category during different periods, instead of the deal count. Apart from the above variables, two variables are included here: *Subtype* (stage of the financing cycle for the target company) and *DevelopmentPhase* (The development stage of the least advanced drug/drug candidate in the portfolio of the target company). Last, we observe that the capital market is expected to show slower and more prolonged reactions to the pandemic due to time lags between investor negotiations and the announcement of deals by target companies. Consequently, we categorized deals announced between 2020 and 2021 as one group and deals announced in 2022 as another group. This setting better characterizes the evolving trends throughout the pandemic.

The descriptive and inferential statistical analyses mentioned in this section were conducted using Python 3.7.

### Regression analyses

Our regression analyses focus on fundraising deals only. Unlike the statistical analyses in the above subsection, here we aggregate deal-level data into category-level data. We categorize deals into 32 groups based on the deal characteristics mentioned above and calculate the total amount raised in these categories in each year-month. Detailed categorization rules are presented in the Results.

Our category-level panel data consists of 32 subjects (unit: category, as defined in the above section) and 144 time points (unit: year-month, from January 2011 to December 2022). Given the panel nature of our data and the inclusion of the time-invariant predictor *Discovery* in our model, we adopt a random effects framework to estimate the association between COVID-19 and the fundraising deal value [27], The regression is specified as follows :

$$Amount_i,_t=\alpha _0 Rate_t + \alpha _1 Early_t + \alpha _2 Late_t + \gamma _0 Discovery_i + \gamma _1 Early_t*Discovery_i$$ $$+\gamma _2 Late_t*Discovery_i + \beta _1 Trend_t + \beta _2 Seasonality_t + u_i + \epsilon _{it}$$

where *Amount* represents the total monthly amount raised in each category (unit: 100 million USD). *Rate* represents the US federal funds rate lagged by three months and is a measure of the global monetary easing environment^5^. The dummy variable *Early* takes the value 1 if the deal was announced between 2020 and 2021, identifying the additional stimulating effect of the pandemic outbreak on the market. A significant positive coefficient for *Early* means the deals announced in 2020-2021 were indeed higher in value, after controlling for the effect of the Fed rate. Similarly, the dummy variable *Late* equals 1 if the deal was announced during 2022. A significant negative coefficient for *Late* suggests the additional drop in the deal amount in the late pandemic, probably led by investors’ expectation of a recession or their declined interest in the biopharma sector after the biotech bubble burst. We control for time variation by including a linear time trend and seasonality.

The binary variable *Discovery* is derived from the categorical variable *DevelopmentPhase*, which consists of four categories: Discovery, Preclinical, Clinical, and FDA reviewed/Marketed (see Table 3 for definitions). These categories represent different stages in the drug development process, with Discovery being the earliest phase. The variable *Discovery* takes the value of 1 if the least advanced drug or drug candidate in the portfolio of the target company is under the discovery phase. The discovery phase includes activities such as target discovery, validation, and the identification of potential drug candidates or lead compounds. It is characterized by higher levels of risk and uncertainty. The increase in discovery-phase investments indicates a greater emphasis on early-stage innovation - by directing resources toward the discovery phase, companies demonstrate their commitment to advancing innovative drug candidates and driving early-stage research. We include two interaction terms to examine if the discovery-phase deal amounts excessively increased during 2020-2021 or 2022, as indicated by the significance of $\gamma _1$ or $\gamma _2$.

The individual-specific random effects, $u_i$, are assumed to be independently and identically distributed and follow a normal distribution $N(0,\sigma _u^2)$. $\epsilon _{it}$ is the random error term, assumed to be uncorrelated with other independent variables and $u_i$. $\epsilon _{it}$ are independently and identically distributed with a normal distribution $N(0,\sigma _\epsilon ^2)$. The random effects modeling presumes that the unobserved differences across deal categories have some influence on the deal amount but are not correlated with the predictors, which is a strong assumption. Hence, we conducted the LM test, which confirmed that the random effects regression is a suitable choice over a simple OLS regression for our analysis ($p<0.001$) [28] .

**Fig. 1:**
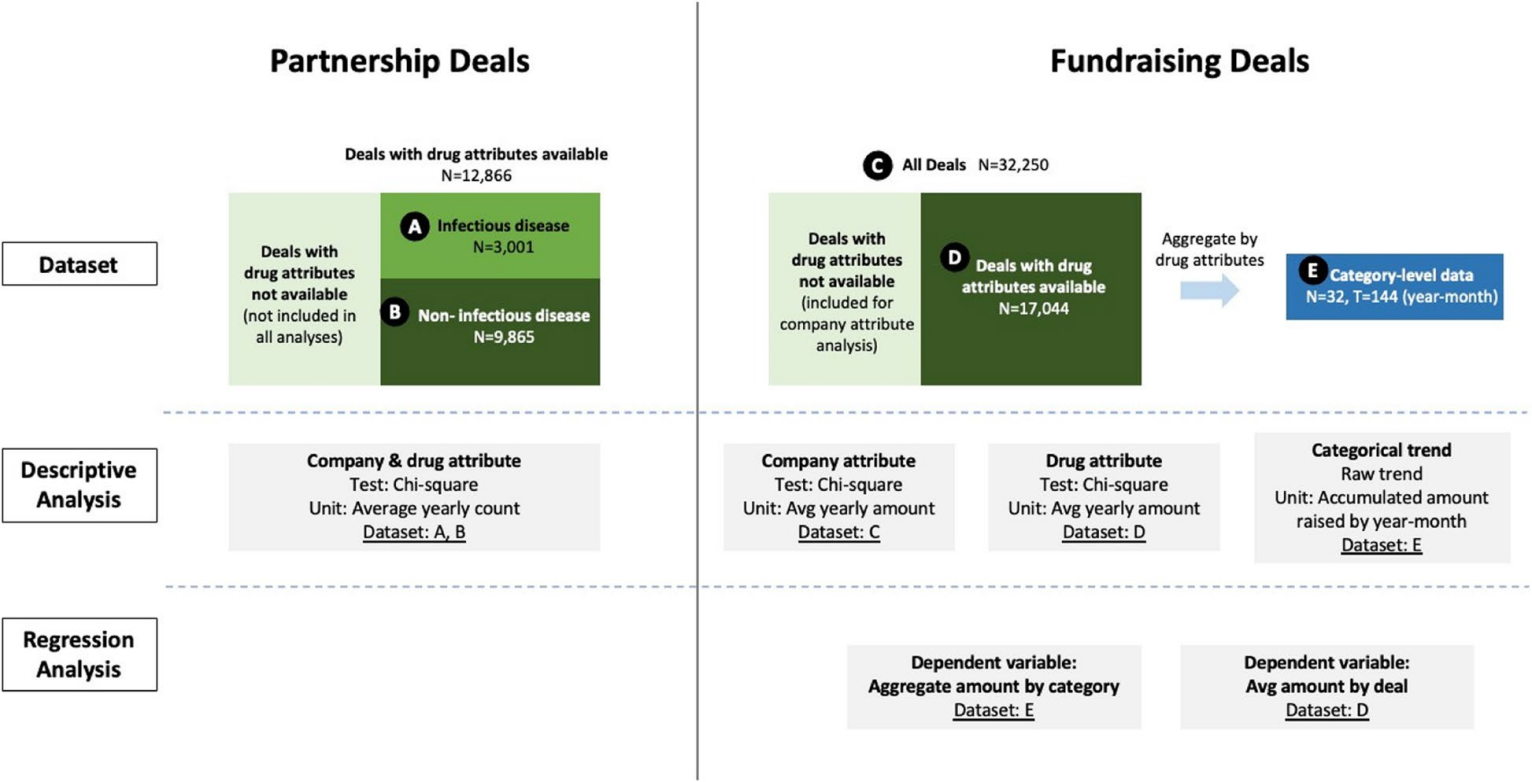
Data sets and methods. Figure 1 visualizes our research flow and provides detailed information about our data sets

Note that the analyses here provide only correlational insights rather than causal inferences. Because monetary policy-making is closely related to the pandemic’s evolution, we cannot perfectly purify each effect. All regression analyses mentioned in this section were conducted using Stata SE, version 17. Figure 1 visualizes our research flow to facilitate understanding.

## Results

### A temporal surge of infectious-disease partnership activities

**Fig. 2:**
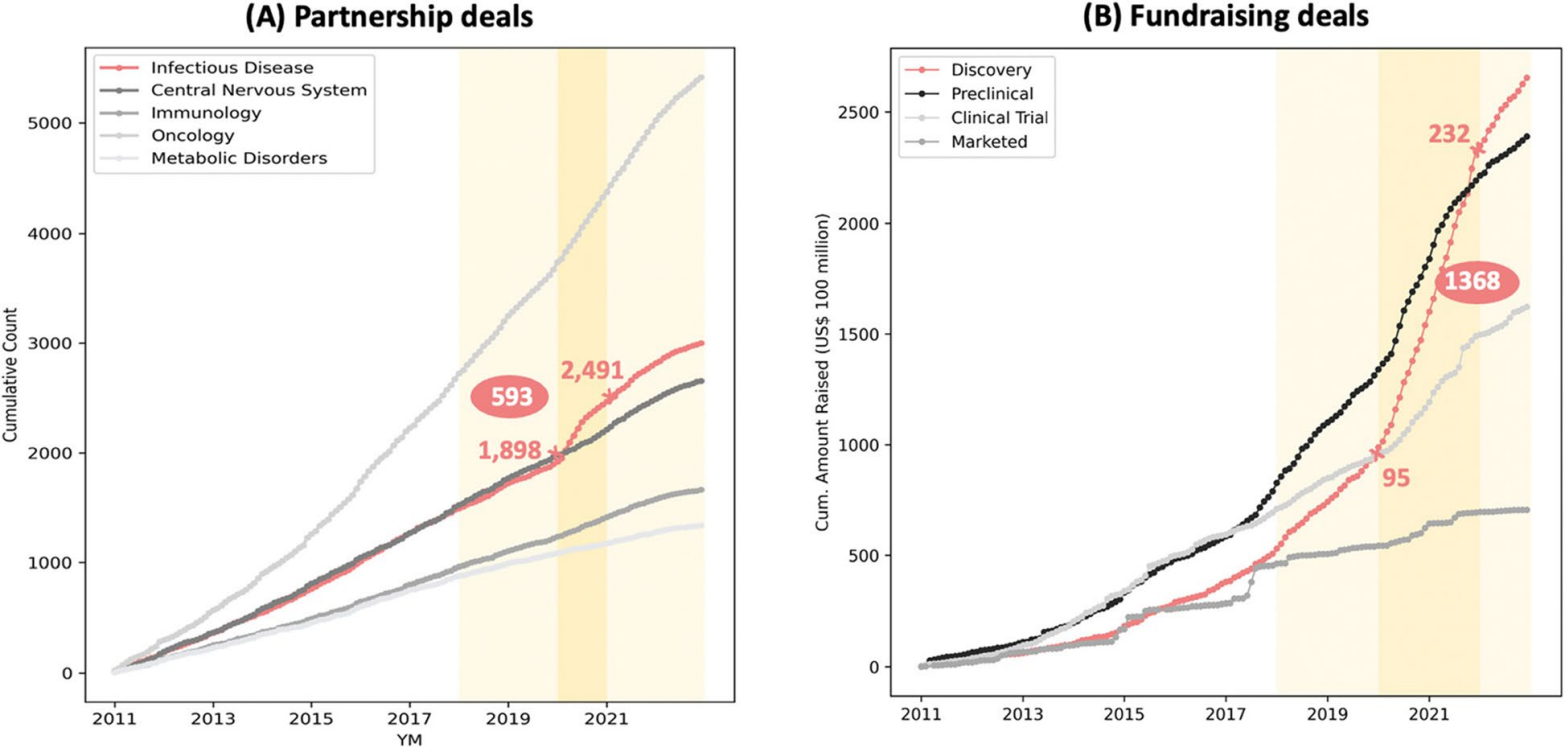
Cumulative Count/ Amount of the Partnership and Fundraising Deals (Monthly). Figure 2(A) shows the cumulative count of partnership deals since January 2011 by different disease types. Note that not all deals are presented in the charts. We pick only deals in the 5 major disease therapy categories for simplicity because the numbers of deals and average deal sizes in other categories are relatively trivial. The light yellow area indicates the main time period of our descriptive analysis (2018-2022). The darker yellow area represents the early phase of the pandemic (2020 alone), during which partnership deals experienced a significant increase. Similarly, Fig. 2(B) displays the cumulative amount of fundraising deals by development phase. The light-yellow area indicates the main time period of our descriptive analysis (2018-2022). In contrast to Fig. 2(A), the darker yellow area corresponds to the period between 2020 and 2021, during which fundraising deals observed a notable increase. In both Fig. 2(A) and 2(B), the pink numbers indicate the label of the corresponding time-points, while the white numbers on pink-filled circles represent the differences between the two time-points. As these are cumulative charts, the white numbers signify the number of deals during the darker-yellow periods

Figure 2(A) provides a quick glimpse of the most important trend in partnership activities, showing the temporal surge in infectious-disease deals in 2020 ($N=518$)^6^. Tables 1 and 2 report the exact number and percentage of average yearly partnership count in the past, before, during, or after 2020. The significance of chi-squared statistics in the tables indicates the composition of partnership activities changed throughout the pandemic, compared with the baseline period (2018-2019).

Diving into these infectious-disease partnership alliances (Table 1), we see that the increase in vaccine co-development programs was significant and persisted throughout 2020-2022 ($p<0.01$). Yet these alliances for vaccine development were mainly used for COVID solutions, suggesting no obvious spillover effects on other infectious disease indications. We observe a significant shift in the formation of alliances - fewer alliances involved public companies only and more alliances formed between public/private companies and institutions ($p<0.05$). These institutions involved - including non-profit foundations, universities/ research institutes, and government agencies - provided funds and research capacity for the co-development alliances. Nonetheless, the collaboration between institutes and the industry did not become the new normal after the outbreak. North America dominated infectious-disease partnership activities during 2020 with $39\%$ of alliances ($N=175$) formed within North America; more alliances involved countries in Asia-Pacific after 2020 yet the change in the composition of geographical regions was not significant.

On the other hand, the yearly count of non-infectious disease partnerships increased by about $15\%$ in 2020 (from 790 to 910), as Table 2 shows. The percentages of partnerships in most therapeutic areas remained almost the same after the pandemic. A potential explanation for the decline in partnerships related to gastrointestinal and central nervous system diseases in 2020 could be that the heightened emphasis on infectious diseases temporarily diverted collaboration resources from developing solutions for other disease areas.

Based on the analysis of Tables 1 and 2, we find no persistent changes in the composition of partnership activities following the pandemic outbreak, except for a notable increase in partnerships specifically related to COVID-19 solutions.

### A focus on innovation in the capital market

Table 3 summarizes how the cumulative amount raised in different types of deals changed throughout the pandemic. Similar to the trends in partnership activities, the shift in focus to infectious disease companies was significant during 2020-2021 ($p<0.05$) but the trend did not last for long. Overall, oncology investments remained its pre-pandemic trend and dominated the fundraising market.

Interestingly, we observe more investments in rising molecule technologies such as Biologic technologies ($p<0.001$), while the growth in Small-Molecule therapies remained stagnant during the period of 2020-2021. Small molecule drugs have traditionally been the predominant therapeutic class due to their ease of synthesis and large-scale production. The shift in focus from this established therapeutic approach to novel biologic technologies or gene/cell therapy signifies an innovative trend. However, this shift in focus appears to be temporary, as indicated by the composition of the deals announced in 2022.

Figure 2(B) presents the most notable and persistent change in the capital market, the growing investments in firms with discovery-phase drugs. This marks an emerging interest in early innovation investments. The trend is observable for almost all financing deals (see eFigure 1 in the supplement). This observed result is also supported by statistical tests provided in Table 3 ($p<0.001$).

Public equity deals (secondary offering and private investment in public equity) used to dominate other biopharma deals. Compared to the pre-pandemic period ($58\%$), they accounted for a smaller proportion of the total investment amount during 2020 and 2021 ($53\%$); however, while most other funds witnessed a bounce-back in 2022, the amount raised in public offering deals remained at a high level and accounted for $68\%$ of the total biopharma investments. $80\%$ of these public offering deals were made in the US; henceforth, eFigure 2 also shows a higher percentage of North American deals in 2022. This reflects the continued expansion of public biopharma firms during the late pandemic. On the other hand, although the amount raised by early- and later-stage VCs did not remain on the peak, these ventures still received more capital than they did during the pre-pandemic period. This suggests a persistent preference for venture investing, a possible indicator of risk-takingness and innovation. Noteworthy, we observe a growing proportion of venture deals in Asia-Pacific during COVID-19 and beyond (eFigure 2).

### Investment growth drivers: discovery-phase COVID-fighting technologies

Since companies have more than one drug in their portfolio, each company could be categorized into several therapy areas or molecule types. The analyses above cannot explicitly capture how investments were distributed across therapy areas/ molecule types. For example, we cannot identify whether the growth of oncology deals during the pandemic indeed reflected more investments in oncology – or it is driven by increasing investments in companies focusing on both oncology and infectious diseases.

**Fig. 3:**
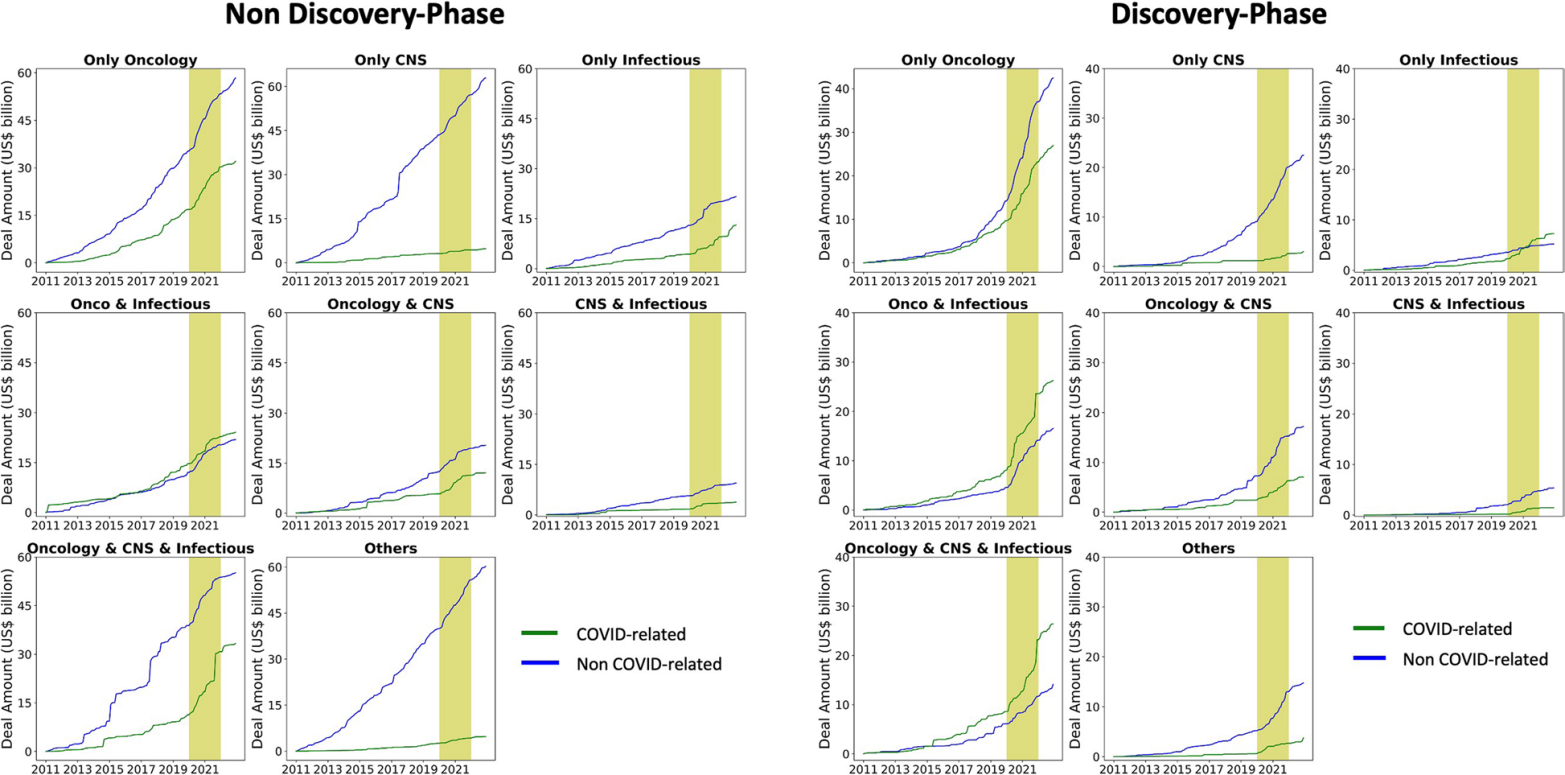
Trends in the 32 categories of fundraising deals by therapy area, molecule type, and development phase. Figure 3 presents the cumulative amount raised in each category since January 2011

To illustrate how the funds are allocated across therapeutic areas, we divide the deals into mutually exclusive and collectively exhaustive groups based on whether the deal involves one of the top 3 therapy areas (oncology, central nervous system (CNS), and infectious disease). Every deal belongs to one of the $8 (= 2^3)$ therapy-area groups. Furthermore, we add another two dimensions to our classification rule: molecule type and development phase. We first split each group into two subgroups based on whether the deal involves COVID-19 solution-related molecule types (vaccine, anti-body, and oligonucleotide^7^[29–31]. We then split each subgroup into two sub-subgroups again based on the deal’s development phase (discovery phase or not). In the end, we have 32 mutually exclusive and collectively exhaustive groups. Figure 3 presents the trend in the total amount raised in each group. Looking at our sample from this different angle, we observe two additional insights. First, deals involving infectious disease and oncology drugs demonstrated higher growth during the pandemic. Second, discovery-stage and COVID-solution-related deals were the growth drivers of infectious disease investments.

### Emerging discovery-phase investments despite interest rate hikes

The deal amount surge was not only led by the COVID-19-driven fundamental factors but also greatly affected by the global monetary easing policies that took place mostly at the same time as the global pandemic hit. Likewise, ater in 2022, the Fed rate started to hike, making it difficult to distinguish whether the deal amount decrease was simply the result of the monetary policies.

**Fig. 4:**
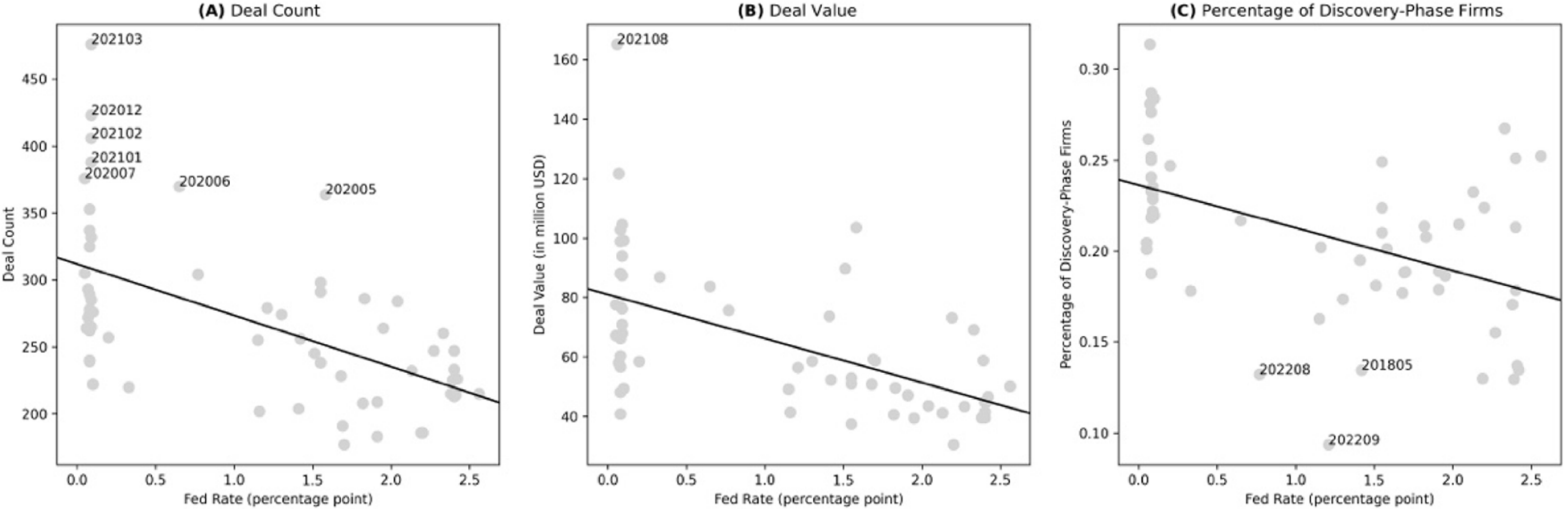
The correlation between the US Fed rate and the monthly biopharma deal characteristics, 2018-2022. These panels demonstrate the relationship between the US Fed fund rate and the deal count/value and the percentage of discovery-phase deals in each year-month. We remark the months in which the count, value, or percentage is abnormally high or low

Figure 4(A-B) shows that the US Fed rate (lagged by three periods) negatively correlated with the deal volumes and values during 2018-2022. Interestingly, we observed two waves of deal volume surges that were not driven solely by the interest rate: May – July 2020 and Dec 2020 – Mar 2021. These time points coincide with the timing of the initial pandemic outbreak and vaccine rollout, suggesting public attention increased the number of biopharma companies invested. However, this attention did not seem to bring more capital to a single company, as there are less extreme deal values that the interest rate could not explain during the early pandemic. Figure 4(C) shows that the percentage of discovery-phase investments also negatively correlated with the Fed rate.

In light of the above illustrative analysis, we conduct category-level regression analyses to decompose the effect of the pandemic itself and the dramatic monetary policies on biopharma deal activities (see Table 4). Most importantly, we investigate the persistent effect of the pandemic on biopharma early innovation - we examine whether discovery-phase deals had raised more funds during COVID-19 and beyond, after controlling for the Fed rate’s effects. From the random effects model, a 1 percentage point increase in the Fed rate was associated with about a 22.2 million-dollar decrease in the deal amounts ($\beta =-0.222, SE=0.08, p<0.001$). The significantly positive coefficient for *Early* suggests an extra stimulating effect on the deal amount that the decreased Fed rate could not explain – ceteris paribus, the deal amount raised in each group was 92.7 million-dollar higher than during 2020 and 2021 ($\beta =0.927, SE=0.24, p<0.001$). On the contrary, the negative coefficient for *Late* indicates that, after considering the Fed rate’s stifling effect on fundraising activities, the biopharma financing deal amount still plummeted by a further of 141.2 million dollars in 2022 ($\beta =-1.412, SE=0.29, p<0.001$). Noteworthy, deals involving discovery-phase drugs had raised more funds during 2020-2021 ($\beta =0.994, SE=0.25, p<0.001$) and did not suffer from an obvious bounce back in 2022 ($\beta =1.206, SE=0.34, p<0.001; 1.206 - 1.412 = -0.206$). This suggests a persistent effect of the pandemic on early innovation biopharma.

Column 2 shows a similar regression specification that uses the average deal value as the dependent variable, based on the unaggregated deal-level sample. The major difference in this setting is the insignificant coefficient for *Early*. This confirms our hypothesis that the soaring investment amount in the early pandemic was driven by the increased deal count, rather than the increased average deal value (Fig. 4).

### Robustness tests

We conduct five sets of robustness tests, assessing the validity of the observed “excessive” effects attributed to the shocks introduced by the pandemic and the subsequent monetary-easing policies, in comparison to the “usual” effects observed in normal circumstances prior to the pandemic. We aim to ensure the coefficients for *Early*, *Late*, $Early*Discovery$, and $Late*Discovery$ were not solely influenced by a mischaracterization of the normal relationship between the Federal funds rate and deal amounts.

For example, the significant coefficient for *Early* might arise from an erroneous model specification, where the assumed linear relationship between the rate and the deal amount does not hold in reality. In such a scenario, the coefficient for *Early* simply captures the unspecified effects of the rate under normal circumstances. That is, the surge in biopharma deal amounts during the early pandemic was exclusively influenced by the hike in the Federal funds rate, just as it tended to be in pre-pandemic conditions.

To address the alternative explanations, we first split our category-level samples into pre-pandemic (2011-2019) and during-pandemic (2020-2022) groups. We run subsample regressions to estimate the relationship between the Fed rate on deal amounts during different periods (see columns (1) and (2) in eTable 1 in the supplement). The estimated coefficient for Rate was insignificant during 2011-2019 and became significant during 2020-2022, after controlling for a linear time trend. This finding suggests that the biopharma sector exhibited a heightened sensitivity to the financial conditions encountered during the pandemic, surpassing what would typically be anticipated in normal circumstances. This overreaction could be attributed to investors responding excessively to the unprecedented and abrupt fluctuations in the Federal funds rate. Another contributing factor is that the biopharma companies’ savior role during the early stages of the pandemic has attracted a surge of interest and investment from investors, yet the investment bubble in the sector did not persist throughout the entire pandemic and experienced a revision in 2022.

In the second and third sets of robustness tests, we introduce alternative specifications to examine the relationship between the Federal funds rate and the deal amount. Specifically, we explore quadratic, square-root, and exponential relationships (columns (3)-(5)), as well as different lag periods for the Federal funds rate (columns (6)-(11)). While the coefficient estimates for the variables *Early* and *Late* exhibit some variation across these specifications, it is noteworthy that all the coefficients maintain the same direction and remain statistically significant at the 0.1% level. This consistency, in conjunction with the earlier test results presented in columns (1) and (2), further strengthens the robustness of the coefficients for the variables *Early* and *Late*. These coefficients effectively capture the exaggerated response of investors and businesses to the financial conditions experienced during the pandemic, surpassing the reactions observed in previous normal circumstances. Most importantly, under all specifications, the coefficients for $Early*Discovery$ and $Late*Discovery$ remain stable, confirming our estimation of the additional growth in discovery-phase deals.

In the fourth set of tests, we investigate whether the heightened emphasis on discovery-stage deals can be attributed solely to the monetary-easing environment. We include a new interaction term between the variables *Rate* and *Discovery* in our model (column (12)). Notably, the coefficients of the original variables, namely $Early*Discovery$ and $Late*Discovery$, remain unaffected by including the new interaction term. The signs of these coefficients remain consistent and statistically significant at the 0.1% level. This result implies that factors beyond financial conditions, such as other fundamental changes stemming from the pandemic, have played a significant role in the increased investments observed in the discovery stage.

We proceed to address concerns regarding the possibility that the coefficients for the variables $Early*Discovery$ and $Late*Discovery$ merely reflect a long-term time trend characterized by an increasing emphasis on discovery-stage investments over time. To examine this, we introduce an interaction term between the variable *Rate* and a linear or squared time trend. Similar to the previous tests, we find that the newly included interaction terms exhibit statistically insignificant signs, while the coefficients for the original variables remain unaffected. This reaffirms the evidence of an intensified focus on discovery-stage investments, which may have been further accelerated by the impact of the pandemic.

In summary, these tests suggest the coefficients for the key coefficients to be robust, indicating that: (1) The biopharma sector demonstrated an amplified sensitivity to the financial conditions experienced during the pandemic, surpassing what would typically be expected in normal circumstances. This heightened response can be attributed to investors reacting excessively to the unprecedented and sudden fluctuations in the Federal Funds rate, which led to significant fluctuations in biopharma deal amounts. (2) The increased focus on discovery-stage investments cannot be attributed to the fluctuation in the Fed rate, suggesting the pandemic has contributed to the intensified emphasis on discovery-stage investments in the biopharma sector.

## Discussion

Based on a data-driven approach, our study quantifies the increase in the level of partnerships and fundraising activities throughout the pandemic. Despite the rapid and prominent increase in COVID-19-related partnerships, we observe no enduring shifts in focus toward other therapy areas and molecule technologies. This empirical evidence is inconsistent with the hypothesis that COVID-19 caused a paradigm shift in partnership alliances and generated spillover effects in the co-development processes for other drugs [11, 13, 14]. Our data shows that the co-development of COVID-19 solutions mostly involved contributions from privately owned businesses and research institutes^8^. This corroborates past literature [34–36] and reinforces the vital role of these relatively smaller entities in generating breakthroughs when faced with unforeseen diseases, prompting support for maintaining their research capacity and capability to prepare for future global health crises.

In the capital market, the need for scientific innovation during the COVID-19 outbreak fueled investors’ interest in biopharmaceuticals. However, it did not last throughout 2022. In addition to the Fed rate’s marginal effect on the deal amount, the amount raised in non-discovery-phase deals excessively decreased in the face of the historical Fed rate hikes in 2022. This could result from the fading interest in biopharma after the initial pandemic outbreak fades and the expectation for an economic downturn. Specifically, the COVID-19 pandemic contributed to the increased venture financing last year, with many biotech ventures going public with high stock prices that have since plummeted. Investors seemed to be more selective in their biotech investments in the late pandemic.

The silver lining is that the discovery-phase investments have shown remarkable growth throughout 2020-2022, despite the higher risks associated with such investments. Interestingly, the traditional economic theory suggests that an increase in interest rates discourages risk-taking investments [37]. However, during 2022, investors did not withdraw their capital from discovery-phase investments in favor of safer alternatives, indicating a sustained focus on early-stage innovation in the biotechnology industry. While our study does not establish a causal relationship, our findings and robustness checks are consistent with the existing literature, which highlights the pandemic’s role in driving increased attention and investment in early-stage innovation. The urgent demand for treatments and vaccines has led to regulatory flexibility, collaborations, the repurposing of existing drugs, and the wider application of advanced technologies, all contributing to higher success rates in early-stage innovation [38–40]. Given these advancements, it is understandable that investors are more inclined to invest in discovery-phase opportunities.

As innovation increases, so does competition for clinical-trial sites and investigator capacity. Future studies could shed light on the post-pandemic trends in clinical trial activities. This would inform entrepreneurs on clinical operations, helping decision-makers to derisk clinical development to get through this unprecedented economic winter [41].

### Limitation

This study has several limitations. First, our sample of partnerships and capital-raising deals is retrieved from a commercial database and does not necessarily reflect all deal activities in the real world. Specifically, the data completeness could systematically vary during the period between 2011 and 2022 due to differential reasons for deal reporting each year. This would potentially jeopardize the validity or completeness of our analyses. Although we have scrutinized the representativeness of the dataset, better deal samples could be sought in the pursuit of more accurate estimates. Second, our regression models for deal value lack important variables (e.g., company age and revenue or other macroeconomic variables) due to the unavailability of relevant data. The exclusion of these variables might lead to omitted variable bias. Last, our inference is suggestive since the data provide only correlational insights instead of causal estimates. It has been only three years since the pandemic began, making it challenging to design natural experiments or find suitable instrumental variables as other economic studies focused on event evaluations usually do. Future research could use other econometric tools for causal inference.

## Conclusion

After a surge in co-development and capital-raising activities during the early pandemic, the partnership counts and fundraising deal amounts bounced back in 2022. Nonetheless, we observe enduring early-innovation investment growth despite the potential economic downturn led by the global monetary-tightening environment. This silver lining in the biopharma sector could inform entrepreneurs of the importance of early innovation and strategic redirection. Our study suggests the need for policy interventions in financing private/public co-development partnerships and non-COVID-related technologies, to maintain their research capacity and generate breakthroughs when faced with unforeseen diseases.

### Supplementary Information

**Additional file 1.** Online Supplement. This file includes all supplemental tables and figures.

## Data Availability

Our primary dataset is not publicly accessible but available by subscribing to GlobalData commercial database; relevant analytic code is available here.

## Footnotes

1 Assembled from public information, GlobalData database provides comprehensive partnership and financial deal information. Each deal is linked to its target companies, which are defined as companies being invested in (private equity/venture capital), companies raising capital (equity or debt offering), or partners to deal (partnerships). The Drugs, Companies, and Financials databases are intercorrelated. The Deals database triggers updates to the respective company profiles in the Companies database, drug profiles in the Drug databases, and Financials database. GlobalData provides an integrated solution so that users can merge these databases using the unique ID of each deal.

2 Most of our analyses focus on data from 2018 to 2022. As our main goal is to compare the trends before and after the pandemic, data from the 2011-2017 period would be irrelevant to our topic. We include data from this distant period to demonstrate that the observed trends after the pandemic was not a reflection of the ongoing long-term trends

3 The is a simple method to control the family-wise error rate. When all null hypotheses are true, the method provides familywise error control that is exact for tests that are stochastically independent. This method is conservative for tests that are positively dependent, which is most likely to be the case in our analysis.

4 Since our sample contains missing deal values (around $15\%$), this total amount is calculated by multiplying each group’s average deal value and deal count instead of simply summing up the values of deals under the same category.

5 We incorporate a lag of three months for the Fed funds rate variable, as the impact of monetary policies on fundraising activities is not immediate. Additionally, in our robustness tests, we explore different lag periods for the rate variable to examine the sensitivity of our results to the choice of lag.

6 The monthly partnership counts fluctuated greatly throughout the twelve-year period. Therefore, we use a cumulative line chart as opposed to a regular line chart to make the time-series patterns look smooth, enhancing data readability. The original monthly counts (non-cumulative) are provided in the online appendix (see Figure Raw Data).

7 Each of the three technologies plays a distinct role in combating COVID-19 and has garnered attention during the pandemic. Vaccines provide the immune system with instructions to produce a harmless piece of the SARS-CoV-2 virus, allowing the immune system to generate a response without the risk of severe disease. Antibodies are proteins produced by the immune system to neutralize pathogens such as viruses. In the context of COVID-19, monoclonal antibodies have been developed to specifically target the SARS-CoV-2 virus. Oligonucleotides are short DNA or RNA molecules. They can be designed to interfere with the genetic material of the SARS-CoV-2 virus, preventing it from replicating and spreading in the body. For instance, antiviral drugs based on small interfering RNA (siRNA) can be developed to target and degrade the virus’s RNA. Furthermore, oligonucleotides are used in the design of mRNA vaccines.

8 The development of the four most common SARS-CoV-2 vaccines–AstraZeneca, Johnson & Johnson, Moderna, and Pfizer-BioNTech–represents the epitome of this phenomenon. Each is a product of collaboration between science and industry or small biotech and big pharma [32, 33].
