# Supplementary material for: Biopharma innovation trends during COVID-19 and beyond: an evidence from global partnerships and fundraising activities, 2011-2022
Source: Global Health. 2023 Aug 14;19:57. doi: 10.1186/s12992-023-00953-6 (PMC10426226; doi:10.1186/s12992-023-00953-6)
Supplement: Supplementary file 1 — Additional file 1. Online Supplement. This file includes all supplemental tables and figures. [file 12992_2023_953_MOESM1_ESM.docx]

**Biopharma innovation during COVID-19 and beyond:**

**Evidence from global partnerships and fundraising activities, 2011-2022**

Tzu-Hui Yu, Yung-Yu Mei, and Yufeng Jane Tseng

**eTable 1. Robustness checks.**

| Coefficient  (Standard error, SE) ^a^ | Subsample regression | | Robustness II:  Nonlinear setting | | Robustness III:  Different lagged terms | | | | | Robustness IV:  Interaction w/ *Discovery* | |
| --- | --- | --- | --- | --- | --- | --- | --- | --- | --- | --- | --- |
|  | 2011-2019 | 2020-2022 | Squared | Sqr. Root | Lag 1 | Lag 2 | Lag 4 | Lag 5 | Lag 6 | Fed rate | Time trend |
|  | (1) | (2) | (3) | (4) | (5) | (6) | (7) | (8) | (9) | (10) | (11) |
| Fed rate | -0.010 | -0.121*** | -0.052*** | -0.620*** | -0.210*** | -0.245*** | -0.204** | -0.198** | -0.230*** | -0.264*** | -0.223*** |
|  | (0.02) | (0.03) | (0.02) | (0.24) | (0.08) | (0.08) | (0.08) | (0.08) | (0.08) | (0.10) | (0.08) |
| Early |  |  | 0.943*** | 0.923*** | 0.898*** | 0.862*** | 0.985*** | 1.021*** | 1.023*** | 0.911*** | 0.959*** |
|  |  |  | (0.23) | (0.24) | (0.24) | (0.24) | (0.23) | (0.22) | (0.22) | (0.24) | (0.25) |
| Late |  |  | -1.393*** | -1.417*** | -1.296*** | -1.398*** | -1.412*** | -1.426*** | -1.491*** | -1.415*** | -1.371*** |
|  |  |  | (0.28) | (0.29) | (0.27) | (0.28) | (0.29) | (0.30) | (0.30) | (0.29) | (0.30) |
| Discovery | 0.283*** | 0.502*** | 0.110 | 0.112 | 0.108 | 0.106 | 0.113 | 0.116 | 0.117 | -0.060 | 0.036 |
|  | (0.03) | (0.09) | (0.17) | (0.17) | (0.17) | (0.17) | (0.17) | (0.17) | (0.17) | (0.26) | (0.24) |
| Early x Discovery |  |  | 0.995*** | 0.993*** | 0.994*** | 0.995*** | 0.992*** | 0.993*** | 0.994*** | 1.018*** | 0.896*** |
|  |  |  | (0.25) | (0.25) | (0.25) | (0.25) | (0.25) | (0.25) | (0.25) | (0.25) | (0.34) |
| Late x Discovery |  |  | 1.210*** | 1.204*** | 1.198*** | 1.200*** | 1.208*** | 1.211*** | 1.212*** | 1.191*** | 1.079** |
|  |  |  | (0.34) | (0.34) | (0.34) | (0.34) | (0.34) | (0.34) | (0.34) | (0.34) | (0.45) |
| Fed rate x Discovery |  |  |  |  |  |  |  |  |  | 0.105 |  |
|  |  |  |  |  |  |  |  |  |  | (0.12) |  |
| Time x Discovery |  |  |  |  |  |  |  |  |  |  | 0.000 |
|  |  |  |  |  |  |  |  |  |  |  | (0.00) |
| Constant | 0.172** | 2.989*** | 0.279 | 0.870** | 0.452 | 0.474* | 0.455 | 0.459 | 0.489* | 0.523* | 0.486* |
|  | (0.08) | (0.26) | (0.27) | (0.35) | (0.28) | (0.28) | (0.28) | (0.28) | (0.28) | (0.29) | (0.28) |
| Trend and seasonality | V | V | V | V | V | V | V | V | V | V |  |
| Sigma_u | 0.417*** | 0.548*** | 1.350*** | 1.350*** | 1.350*** | 1.349*** | 1.349*** | 1.350*** | 1.350*** | 1.350*** | 1.350*** |
|  | (0.05) | (0.07) | (0.18) | (0.18) | (0.18) | (0.18) | (0.18) | (0.18) | (0.18) | (0.18) | (0.18) |
| Sigma_e | 0.447*** | 0.616*** | 3.069*** | 3.069*** | 3.071*** | 3.069*** | 3.069*** | 3.069*** | 3.070*** | 3.070*** | 3.069*** |
|  | (0.01) | (0.01) | (0.03) | (0.03) | (0.03) | (0.03) | (0.03) | (0.03) | (0.03) | (0.03) | (0.03) |
| N of cases | 3456 | 1152 | 4608 | 4608 | 4608 | 4608 | 4608 | 4608 | 4608 | 4608 | 4608 |

^a^ *p<0.05; **p<0.01; ***p<0.001.

**eTable 2. Contigency table for 32 categories of fundraising deals by therapy area, molecule type, and development phase**

| **Therapy Area** |  | **Discovery** | | **Non-Discovery** | | **Total** |
| --- | --- | --- | --- | --- | --- | --- |
|  |  | **Vaccine and Antibody** | **Others** | **Vaccine and Antibody** | **Others** |  |
| **Only Oncology** | Amount | 28,419 | 47,474 | 35,508 | 63,166 | 174,567 |
|  | Count | 581 | 1,039 | 827 | 2,092 | 4,539 |
| **Only CNs** | Amount | 3,284 | 24,003 | 6,325 | 68,569 | 102,181 |
|  | Count | 69 | 645 | 104 | 1,939 | 2,757 |
| **Only Infectious** | Amount | 7,690 | 5,588 | 13,239 | 23,341 | 49,859 |
|  | Count | 189 | 298 | 345 | 806 | 1,638 |
| **Oncology & Infectious** | Amount | 29,278 | 17,354 | 27,322 | 23,721 | 97,676 |
|  | Count | 409 | 340 | 473 | 577 | 1,799 |
| **Oncology & CNS** | Amount | 8,125 | 19,069 | 13,522 | 22,118 | 62,834 |
|  | Count | 165 | 337 | 195 | 525 | 1,222 |
| **CNS & Infectious** | Amount | 1,708 | 5,580 | 3,923 | 10,639 | 21,851 |
|  | Count | 40 | 123 | 83 | 353 | 599 |
| **Oncology & CNS & Infectious** | Amount | 27,589 | 16,752 | 35,502 | 59,554 | 139,397 |
|  | Count | 296 | 303 | 436 | 613 | 1,648 |
| **Others** | Amount | 4,032 | 15,866 | 5,085 | 66,611 | 91,595 |
|  | Count | 82 | 518 | 155 | 2,136 | 2,891 |
| **Total** | Total Amount | 110,125 | 151,688 | 140,428 | 337,720 | 739,960 |
|  | Total Count | 1,831 | 3,603 | 2,618 | 9,041 | 17,093 |

# eTable 3. The composition of fundraising activities (average yearly count).

|  |  | **Average yearly count (%)^a^** | | | | | | | | | |
| --- | --- | --- | --- | --- | --- | --- | --- | --- | --- | --- | --- |
|  |  | **2011-2017** | | **2018-2019**  (base group) | | **2020-2021** | | | **2022** | | |
| **All Fundraising Deals (N=32,250)** | | **Avg yearly count: 2,293** | | **Avg yearly**  **count: 2,683** | | **Avg yearly**  **count: 2,615** | | | **Avg yearly**  **count: 1,617** | | |
| **Deal Subtype ^b^** | Stage of the financing cycle for the target company |  |  |  |  |  |  |  |  |  |  |
| Early stage | Conceptualization stage with products not fully developed | 671 | 29% | 724 | 27% | 1,034 | 27% | ** | 1,002 | 32% | *** |
| Later stage | The product is fully developed, tested, and ready to be launched | 543 | 24% | 576 | 21% | 830 | 22% | ** | 664 | 21% | *** |
| Private equity | Acquired by a private equity firm | 131 | 6% | 180 | 7% | 321 | 8% | ** | 239 | 8% | *** |
| IPO | Initial public offering | 104 | 5% | 116 | 4% | 210 | 6% | ** | 117 | 4% | *** |
| Other equity offerings | Private investment in public equity or secondary offerings | 844 | 37% | 1,086 | 40% | 1,410 | 37% | ** | 1,133 | 36% | *** |
| **Region ^b^** | The geographical region where the deal took place |  |  |  |  |  |  |  |  |  |  |
| Asia-Pacific | - | 261 | 11% | 373 | 14% | 838 | 22% | *** | 683 | 22% | *** |
| Europe | - | 428 | 19% | 484 | 18% | 676 | 18% | *** | 485 | 15% | *** |
| North America | - | 1,550 | 68% | 1,764 | 66% | 2,209 | 58% | *** | 1,926 | 61% | *** |
| Others | - | 53 | 2% | 62 | 2% | 82 | 2% | *** | 61 | 2% | *** |
|  | |  | |  | |  | | |  | | |
| **All Fundraising Deals w/ Available Drug Attributes (N=17,044)** | | **Avg yearly count: 1,131** | | **Avg yearly**  **count: 1,574** | | **Avg yearly**  **count: 2,201** | | | **Avg yearly**  **count: 1,551** | | |
| **Development Phase ^b^** | The development stage of the least advanced drug/drug candidate in the portfolio of the target company |  |  |  |  |  |  |  |  |  |  |
| Discovery | Identification and optimization of a substance for therapeutic use | 314 | 28% | 484 | 31% | 890 | 40% | *** | 664 | 43% | *** |
| Preclinical | Testing of a drug in nonhuman animals or in vitro studies for a candidate drug | 456 | 40% | 678 | 43% | 849 | 39% | *** | 535 | 34% | *** |
| Clinical | Testing of a drug in humans to determine health outcomes | 310 | 27% | 372 | 24% | 408 | 19% | *** | 313 | 20% | *** |
| FDA reviewed/Marketed | FDA-reviewed or marketed drugs | 51 | 5% | 40 | 3% | 54 | 2% | *** | 39 | 3% | *** |
| **Therapy Area ^c^** | With a drug/drug candidate in a specific disease area |  |  |  |  |  |  |  |  |  |  |
| Metabolic Disorders | - | 314 | 27% | 380 | 24% | 518 | 23% |  | 358 | 21% |  |
| Cardiovascular | - | 241 | 21% | 262 | 16% | 386 | 17% |  | 265 | 16% |  |
| Immunology | - | 317 | 27% | 453 | 28% | 662 | 30% |  | 444 | 26% |  |
| Oncology | - | 601 | 51% | 862 | 54% | 1272 | 57% | * | 901 | 54% |  |
| Respiratory | - | 225 | 19% | 301 | 19% | 412 | 19% |  | 279 | 17% |  |
| Musculoskeletal Disorders | - | 175 | 15% | 232 | 14% | 348 | 16% |  | 246 | 15% |  |
| Infectious Disease | - | 378 | 32% | 495 | 31% | 770 | 35% | * | 572 | 34% |  |
| Gastrointestinal | - | 251 | 21% | 332 | 21% | 488 | 22% |  | 376 | 22% |  |
| Central Nervous System | - | 420 | 36% | 580 | 36% | 820 | 37% |  | 581 | 35% |  |
| **Molecule Type ^c^** | With a drug/drug candidate of a specific molecule type |  |  |  |  |  |  |  |  |  |  |
| Gene&Cell | - | 149 | 13% | 256 | 16% | 454 | 21% | *** | 324 | 20% | ** |
| Protein | - | 151 | 13% | 173 | 11% | 277 | 13% |  | 167 | 10% |  |
| Vaccine | - | 114 | 10% | 128 | 8% | 226 | 11% | * | 176 | 11% | ** |
| Anti-Body | - | 199 | 17% | 282 | 18% | 472 | 22% | ** | 314 | 20% |  |
| Peptide | - | 124 | 11% | 136 | 9% | 214 | 10% |  | 145 | 9% |  |
| Small Molecule | - | 701 | 60% | 912 | 58% | 1202 | 56% |  | 904 | 57% |  |
| Recombinant | - | 154 | 13% | 161 | 10% | 224 | 10% |  | 153 | 10% |  |
| Biologic | - | 31 | 3% | 67 | 4% | 109 | 5% |  | 96 | 6% | * |
| Oligonucleotide | - | 71 | 6% | 90 | 6% | 163 | 8% | * | 121 | 8% | * |
| a We use the pre-outbreak deals as the baseline group to perform chi-squared tests, examining whether the composition of fundraising activities changed during or after the initial global outbreak. The significance of each chi-squared test is denoted by asterisks (*P<0.05, **P<0.01, ***P < 0.001). No missing values for all variables listed in the table.  b This is a nominal variable, so the percentages of all subjects add up to 100%.  c This is a set of binary variables that are not mutually exclusive and collectively exhaustive. Since companies have more than one drug in their portfolio, each company could be categorized into several therapy areas or molecule types. | | | | | | | | | | | |

# eTable 4. The composition of partnership activities – Multiple testing correction

|  | **Average yearly count (%)^a^** | | | | | | | | | | | | | | | |
| --- | --- | --- | --- | --- | --- | --- | --- | --- | --- | --- | --- | --- | --- | --- | --- | --- |
|  | **2011-2017** | | **2018-2019**  (base group) | | **2020** | | | | | | **2021-2022** | | | | | |
| **Infectious Disease Partnership (N=3,001)** |  |  |  |  |  |  | **Unadjusted** | | **Adjusted** | |  |  | **Unadjusted** | | **Adjusted** | |
|  |  |  |  |  |  |  | **p-val** | **Sig.** | **p-val** | **Sig.** |  |  | **p-val** | **Sig.** | **p-val** | **Sig.** |
| **Molecule Type** |  |  |  |  |  |  |  |  |  |  |  |  |  |  |  |  |
| Gene/Cell Therapy | 9 | 5% | 14 | 7% | 27 | 5% | 0.449 |  | 0.972 |  | 10 | 4% | 0.203 |  | 0.796 |  |
| Protein | 13 | 6% | 11 | 6% | 20 | 4% | 0.376 |  | 0.963 |  | 16 | 6% | 1 |  | 1 |  |
| Vaccine | 43 | 22% | 30 | 17% | 170 | 34% | 0 | *** | 0 | *** | 72 | 29% | 0.003 | ** | 0.027 | ** |
| Anti-Body | 30 | 15% | 48 | 26% | 121 | 24% | 0.65 |  | 0.985 |  | 56 | 22% | 0.412 |  | 0.886 |  |
| Peptide | 12 | 6% | 10 | 5% | 20 | 4% | 0.554 |  | 0.982 |  | 6 | 2% | 0.132 |  | 0.678 |  |
| Small | 98 | 50% | 88 | 48% | 155 | 31% | 0 | *** | 0 | *** | 102 | 41% | 0.226 |  | 0.796 |  |
| Recombinant | 16 | 8% | 18 | 10% | 48 | 10% | 1 |  | 1 |  | 22 | 9% | 0.905 |  | 0.991 |  |
| Biologic | 3 | 2% | 4 | 2% | 9 | 2% | 1 |  | 1 |  | 8 | 3% | 0.667 |  | 0.963 |  |
| Oligonucleotide | 8 | 4% | 14 | 8% | 33 | 7% | 0.78 |  | 0.989 |  | 26 | 11% | 0.352 |  | 0.886 |  |
| **Non-Infectious Disease Partnership (N=9,865)** |  | |  | |  |  |  |  |  |  |  |  |  |  |  |  |
| **Therapy Area** |  |  |  |  |  |  |  |  |  |  |  |  |  |  |  |  |
| Metabolic Disorders | 145 | 41% | 230 | 35% | 106 | 12% | 1 |  | 1 |  | 90 | 11% | 0.539 |  | 0.952 |  |
| Cardiovascular | 116 | 33% | 159 | 24% | 465 | 51% | 0.055 |  | 0.364 |  | 408 | 48% | 0.553 |  | 0.952 |  |
| Immunology | 133 | 37% | 256 | 39% | 57 | 6% | 0 | *** | 0 | *** | 76 | 9% | 0.116 |  | 0.709 |  |
| Oncology | 202 | 57% | 437 | 66% | 49 | 5% | 0.102 |  | 0.529 |  | 48 | 6% | 0.183 |  | 0.77 |  |
| Respiratory | 111 | 31% | 166 | 25% | 63 | 7% | 0.215 |  | 0.766 |  | 63 | 7% | 0.398 |  | 0.952 |  |
| Musculoskeletal Disorders | 103 | 29% | 151 | 23% | 57 | 6% | 0.334 |  | 0.803 |  | 49 | 6% | 0.168 |  | 0.77 |  |
| Infectious Disease | 142 | 40% | 250 | 38% | 54 | 6% | 0.524 |  | 0.892 |  | 42 | 5% | 0.117 |  | 0.709 |  |
| Gastrointestinal | 129 | 36% | 182 | 28% | 160 | 18% | 0.005 | ** | 0.044 | * | 184 | 22% | 0.45 |  | 0.952 |  |
| Central Nervous System | 172 | 49% | 266 | 40% | 51 | 6% | 0.864 |  | 0.982 |  | 42 | 5% | 0.408 |  | 0.952 |  |
| **Molecule Type** |  |  |  |  |  |  |  |  |  |  |  |  |  |  |  |  |
| Gene/Cell Therapy | 93 | 13% | 140 | 18% | 170 | 20% | 0.512 |  | 0.973 |  | 131 | 17% | 0.479 |  | 0.962 |  |
| Protein | 51 | 7% | 36 | 5% | 41 | 5% | 1 |  | 1 |  | 39 | 5% | 0.831 |  | 0.995 |  |
| Vaccine | 23 | 3% | 22 | 3% | 18 | 2% | 0.386 |  | 0.967 |  | 12 | 2% | 0.128 |  | 0.666 |  |
| Anti-Body | 128 | 18% | 146 | 19% | 178 | 21% | 0.453 |  | 0.973 |  | 175 | 23% | 0.11 |  | 0.65 |  |
| Peptide | 38 | 5% | 32 | 4% | 36 | 4% | 1 |  | 1 |  | 30 | 4% | 0.939 |  | 0.996 |  |
| Small Molecule | 362 | 51% | 355 | 47% | 369 | 43% | 0.152 |  | 0.733 |  | 350 | 45% | 0.584 |  | 0.97 |  |
| Recombinant | 42 | 6% | 27 | 4% | 17 | 2% | 0.075 |  | 0.504 |  | 19 | 2% | 0.266 |  | 0.885 |  |
| Biologic | 13 | 2% | 15 | 2% | 17 | 2% | 1 |  | 1 |  | 22 | 3% | 0.31 |  | 0.892 |  |
| Oligonucleotide | 27 | 4% | 28 | 4% | 36 | 4% | 0.739 |  | 0.995 |  | 29 | 4% | 1 |  | 1 |  |
| a We use the pre-outbreak deals as the baseline group to perform chi-squared tests, examining whether the composition of partnership activities changed during or after the initial global outbreak. The significance of each chi-squared test is denoted by asterisks (*P<0.05, **P<0.01, ***P < 0.001). | | | | | | | | | | | | | | | | |

# eTable 5. The composition of fundraising activities – Multiple testing correction

#

|  | **Average yearly amount – in US$ 100 million dollars (%)^a^** | | | | | | | | | | | | | | | |
| --- | --- | --- | --- | --- | --- | --- | --- | --- | --- | --- | --- | --- | --- | --- | --- | --- |
|  | **2011-2017** | | **2018-2019**  (base group) | | **2020** | | | | | | **2021-2022** | | | | | |
| **All Fundraising Deals w/ Available Drug Attributes (N=17,044)** |  | |  | |  |  | **Unadjusted** | | **Adjusted** | |  |  | **Unadjusted** | | **Adjusted** | |
|  |  |  |  |  |  |  | **p-val** | **Sig.** | **p-val** | **Sig.** |  |  | **p-val** | **Sig.** | **p-val** | **Sig.** |
| **Therapy Area** |  |  |  |  |  |  |  |  |  |  |  |  |  |  |  |  |
| Metabolic Disorders | 145 | 41% | 230 | 35% | 498 | 34% | 0.662 |  | 0.662 |  | 212 | 29% | 0.033 | * | 0.264 |  |
| Cardiovascular | 116 | 33% | 159 | 24% | 378 | 26% | 0.461 |  | 0.662 |  | 129 | 18% | 0.006 | ** | 0.066 |  |
| Immunology | 133 | 37% | 256 | 39% | 631 | 43% | 0.093 |  | 0.465 |  | 257 | 36% | 0.256 |  | 0.644 |  |
| Oncology | 202 | 57% | 437 | 66% | 1030 | 70% | 0.096 |  | 0.48 |  | 437 | 61% | 0.041 | * | 0.328 |  |
| Respiratory | 111 | 31% | 166 | 25% | 428 | 29% | 0.067 |  | 0.402 |  | 140 | 20% | 0.015 | * | 0.137 |  |
| Musculoskeletal Disorders | 103 | 29% | 151 | 23% | 381 | 26% | 0.15 |  | 0.6 |  | 140 | 19% | 0.141 |  | 0.605 |  |
| Infectious Disease | 142 | 40% | 250 | 38% | 662 | 45% | 0.003 | ** | 0.033 | * | 284 | 39% | 0.59 |  | 0.644 |  |
| Gastrointestinal | 129 | 36% | 182 | 28% | 453 | 31% | 0.15 |  | 0.6 |  | 182 | 25% | 0.363 |  | 0.644 |  |
| Central Nervous System | 172 | 49% | 266 | 40% | 633 | 43% | 0.263 |  | 0.662 |  | 270 | 38% | 0.333 |  | 0.644 |  |
| **Molecule Type** |  |  |  |  |  |  |  |  |  |  |  |  |  |  |  |  |
| Gene&Cell | 45 | 13% | 119 | 18% | 357 | 25% | 0.001 | *** | 0.006 | ** | 149 | 21% | 0.162 |  | 0.692 |  |
| Protein | 69 | 19% | 97 | 15% | 308 | 21% | 0.001 | *** | 0.006 | ** | 111 | 16% | 0.692 |  | 0.692 |  |
| Vaccine | 31 | 9% | 52 | 8% | 169 | 12% | 0.014 | * | 0.056 |  | 76 | 11% | 0.087 |  | 0.486 |  |
| Anti-Body | 69 | 19% | 167 | 25% | 506 | 35% | 0 | *** | 0 | *** | 216 | 31% | 0.032 | * | 0.256 |  |
| Peptide | 50 | 14% | 71 | 11% | 190 | 13% | 0.169 |  | 0.338 |  | 67 | 10% | 0.506 |  | 0.692 |  |
| Small Molecule | 241 | 68% | 382 | 58% | 839 | 58% | 0.798 |  | 0.798 |  | 382 | 54% | 0.162 |  | 0.692 |  |
| Recombinant | 73 | 21% | 95 | 14% | 244 | 17% | 0.207 |  | 0.414 |  | 93 | 13% | 0.578 |  | 0.692 |  |
| Biologic | 7 | 2% | 17 | 3% | 107 | 7% | 0 | *** | 0 | *** | 41 | 6% | 0.006 | ** | 0.054 |  |
| Oligonucleotide | 22 | 6% | 47 | 7% | 154 | 11% | 0.019 | * | 0.076 |  | 45 | 6% | 0.592 |  | 0.692 |  |
| a We use the pre-outbreak deals as the baseline group to perform chi-squared tests, examining whether the composition of fundraising activities changed during or after the initial global outbreak. The significance of each chi-squared test is denoted by asterisks (*P<0.05, **P<0.01, ***P < 0.001). No missing values for all variables listed in the table. | | | | | | | | | | | | | | | | |

eFigure 1. The cumulative total value of fundraising deals by deal subtype and development stage (in USD millions)


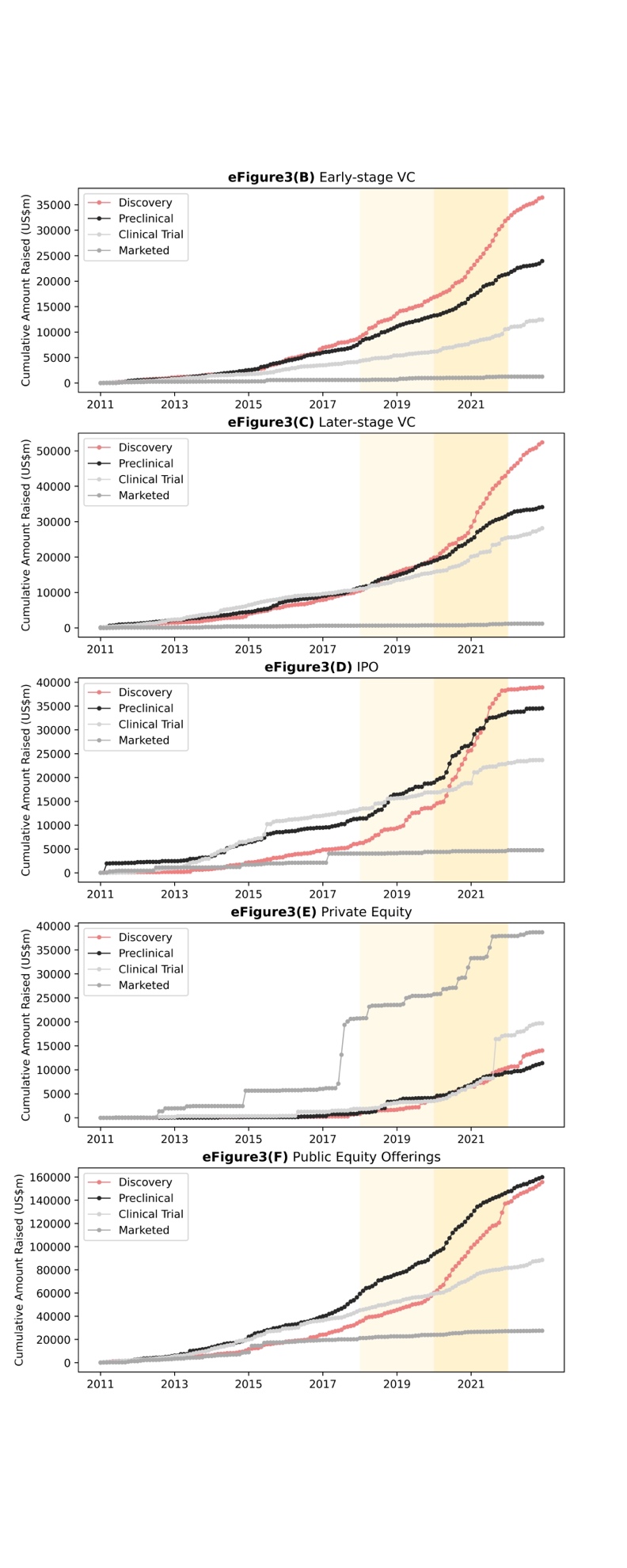


eFigure 2. Geographical Composition of the Fundraising Deals by Fund Types

**
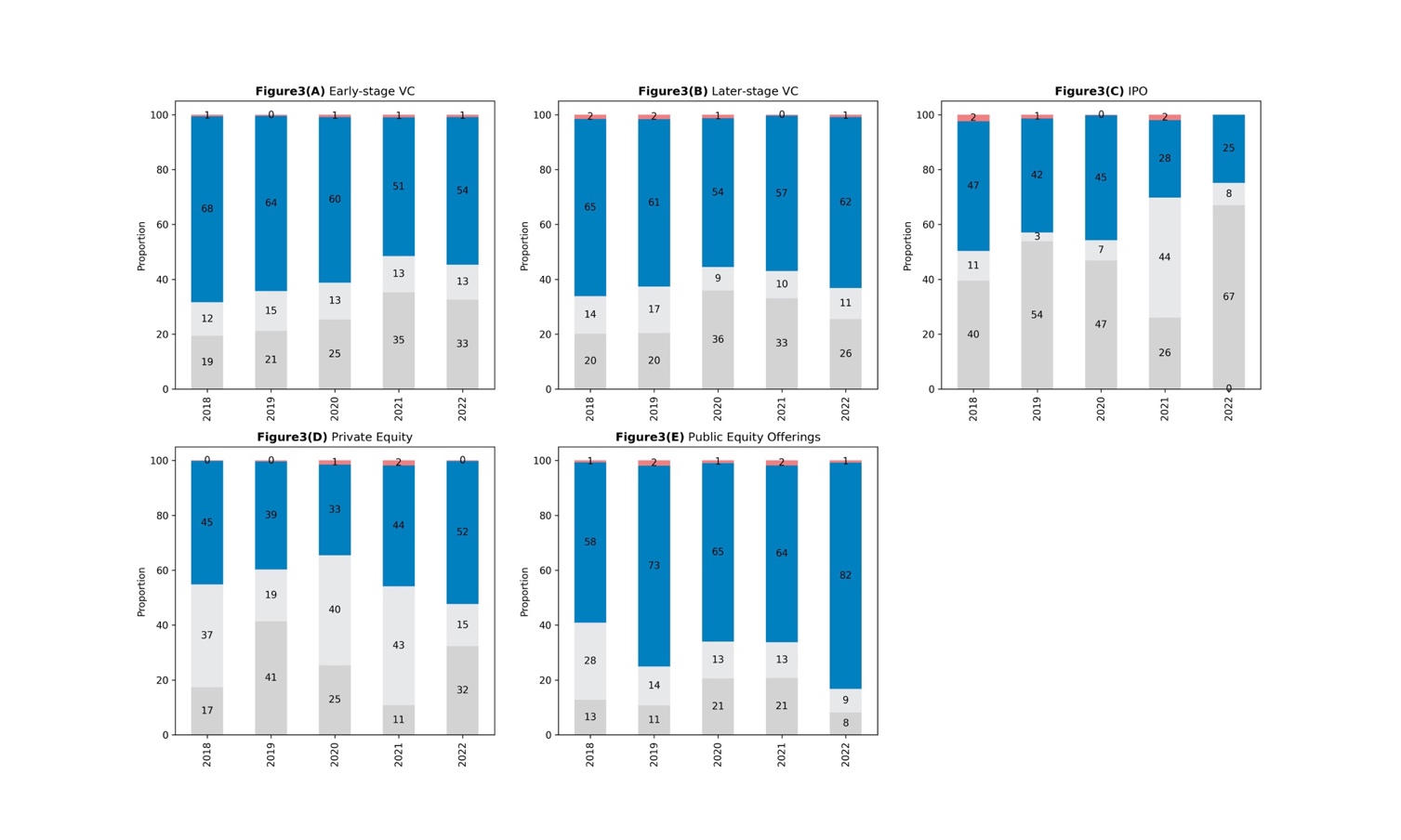
**
